# Metformin reverses prostate cancer resistance to enzalutamide by targeting TGF-*β*1/STAT3 axis-regulated EMT

**DOI:** 10.1038/cddis.2017.417

**Published:** 2017-08-24

**Authors:** Qiuli Liu, Dali Tong, Gaolei Liu, Jing Xu, Khang Do, Kyla Geary, Dianzheng Zhang, Jun Zhang, Yao Zhang, Yaoming Li, Gang Bi, Weihua Lan, Jun Jiang

**Affiliations:** 1 Department of Urology, Institute of Surgery Research, Daping Hospital, Third Military Medical University, Chongqing 400042, China; 2 Department of Bio-Medical Sciences, Philadelphia College of Osteopathic Medicine, 4170 City Avenue, Philadelphia, PA 19131, USA

## Abstract

Although the newly developed second-generation anti-androgen drug enzalutamide can repress prostate cancer progression significantly, it only extends the survival of prostate cancer patients by 4–6 months mainly due to the occurrence of enzalutamide resistance. Most of the previous studies on AR antagonist resistance have been focused on AR signaling. Therefore, the non-AR pathways on enzalutamide resistance remain largely unknown. By using C4-2, CWR22Rv1 and LNCaP cell lines, as well as mice bearing CWR22Rv1 xenografts treated with either enzalutamide or metformin alone or in combination, we demonstrated that metformin is capable of reversing enzalutamide resistance and restores sensitivity of CWR22Rv1 xenografts to enzalutamide. We showed that metformin alleviated resistance to enzalutamide by inhibiting EMT. Furthermore, based on the effect of metformin on the activation of STAT3 and expression of TGF-*β*1, we propose that metformin exerts its effects by targeting the TGF-*β*1/STAT3 axis. These findings suggest that combination of metformin with enzalutamide could be a more efficacious therapeutic strategy for the treatment of castration-resistant prostate cancer.

Prostate cancer is the most commonly diagnosed cancer in men in the western world which causes over 80 deaths per day in the United States of American.^1^ Androgen-deprivation therapy (ADT) has been used as a standard treatment for advanced prostate cancer for more than six decades.^2^ Although the recently developed ADT drugs abiraterone and enzalutamide slow down tumor growth effectively,^3, 4, 5^ recurrence with metastases after ADT are still the major concern in the treatment of castration-resistant prostate cancer (CRPC).^6^ Lin *et al.*^7^ found that bicalutamide and enzalutamide can even induce macrophage migration and tumor cell invasion in orthotopic tumor models of CRPC. Mechanistically, enzalutamide exerts its anti-prostate cancer effects by interrupting the interaction between AR and DHT, blocking AR nuclear translocation, and preventing recruitment of AR to androgen responsive elements.^8^ Nevertheless, enzalutamide is only capable of extending survival of CRPC patients by 4–6 months partially due to the development of enzalutamide resistance^9^ but the underlying mechanisms have not been well defined.

Epithelial mesenchymal transition (EMT) was initially identified as a developmental process from an epithelial phenotype to an invasive mesenchymal phenotype. Many transcriptional factors including Twist, Snail, Slug and Zeb1/2 are involved in this process.^10^ EMT also plays essential roles in cancer cell invasion and metastasis.^10, 11, 12, 13, 14^ In prostate cancer, elevated levels of mesenchymal biomarkers and reduced epithelial differentiation markers are highly correlated with invasion, metastasis and resistance to ADT.^15, 16, 17, 18^ It has also been proposed that ADT itself may exert a causal effect in EMT^19^ and subsequently lead to resistance to enzalutamide treatment.^20, 21^

Metformin possesses anti-tumor effects to many cancers, including melanoma,^22^ colon cancer,^23^ ovarian cancer,^24^ bladder cancer,^25^ prostate cancer.^26^ Different mechanisms have been proposed including inhibiting proliferation, enhancing apoptosis, repressing EMT, targeting cancer stem cells and inhibiting autophagy. Results from a multicenter phase 2 clinical trial showed that metformin could yield objective PSA responses and induce disease stabilization for chemotherapy-naive CRPC.^27^ We found that combinatorial treatment of metformin and bicalutamide can additively repress the growth of prostate cancer.^26^ In this research, we demonstrated that metformin is capable of inhibiting enzalutamide-induced EMT in prostate cancer cells via repressing TGF-*β*1/STAT3 axis.

## Results

### Metformin enhances enzalutamide’s inhibitory effect on prostate cancer cell growth

**Figure 1:**
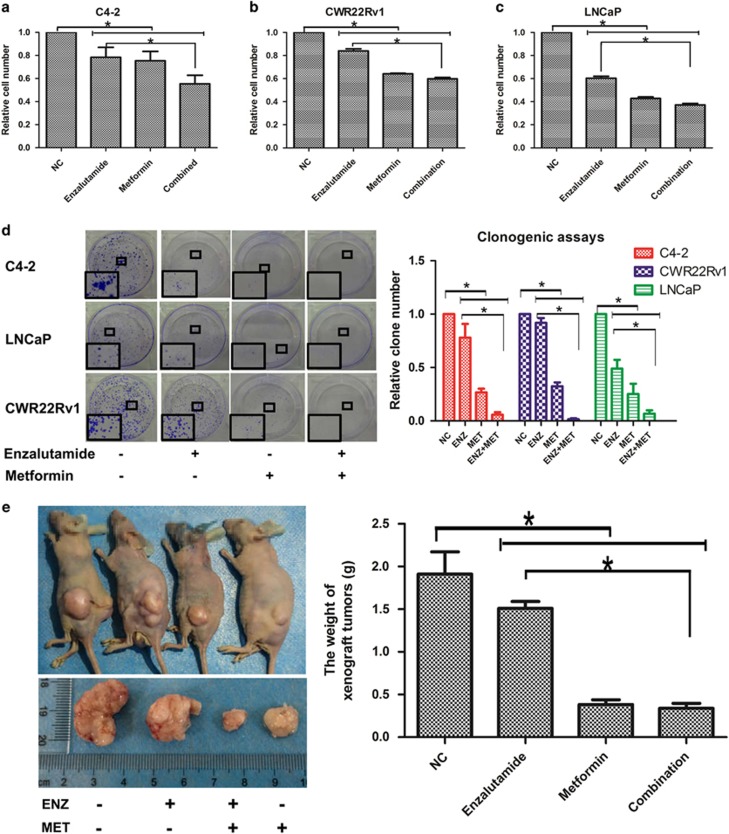
Combinatorial effects of enzalutamide and metformin on prostate cancer growth. (**a–c**) C4-2, CWR22Rv1 and LNCaP cells were seeded in 96-well plates with 0.5 × 10^5^ cells per well in growth media with or without enzalutamide (20 *μ*M) and metformin (5 mM) and cultured for 48 h. Cell viabilities were estimated by CCK-8. (**d**) Those three cell lines were seeded in six-well plates with 1000 cells per well and treated as indicated for 14 days. Cells were fixed with methanol, stained with crystal violet, and the numbers of colonies were counted. (**e**) Mice bearing CWR22Rv1 xenografts were treated with vehicle control, enzalutamide, metformin or their combination for 3 weeks; the tumors were collected and weighed. The data represent means±S.D. *P<0.05

To investigate whether metformin can enhance the anti-tumor effect of enzalutamide on prostate cancer, three prostate cancer cells, C4-2, CWR22Rv1 and LNCaP were treated with either enzalutamide (20 *μ*M) or metformin (5 mM) alone or in combination of both. As shown in Figures 1a–c, comparing to the non-treatment control, the growth rates of all three cancer cell lines were inhibited when they were treated with either agent alone. However, the inhibitive effect is more dramatic when they were combined. In addition, we tested the additive/synergistic effects between enzalutamide and metformin using colony formation assays. Again, colony formation was significantly inhibited for both C4-2 and LNCaP cells when they were treated with either reagent alone, and no colony was detectable when they were treated with the combination of both reagents (Figure 1d). It is well established that CWR22Rv1 cells are resistant to enzalutamide treatment mainly due to the increased expression of the AR-V7, an AR variant constitutively active even in the absence of androgens. To test whether metformin can reverse the resistance of CW22Rv1 cells to enzalutamide, mice bearing xenografts derived from CWR22Rv1 cells were treated with either vehicle, enzalutamide and metformin alone or combination of enzalutamide and metformin for 3 weeks as described in methods. As shown in Figure 1e, the sizes and weights of the tumors were comparable between the enzalutamide-treated and those in the vehicle group and the mouse body weights were comparable in all the four groups. However, metformin alone decreased the sizes and weights of the tumors derived from the CW22Rv1 cells. Surprisingly, the combination of enzalutamide and metformin showed more dramatic inhibitive effect than metformin alone although enzalutamide itself had no effect on the tumors formed from these cells. An alternative explanation is that metformin may be able to reverse the resistance of CWR22Rv1 xenografts to enzalutamide.

### Effect of metformin on enzalutamide-mediated cancer cell invasion and migration

**Figure 2:**
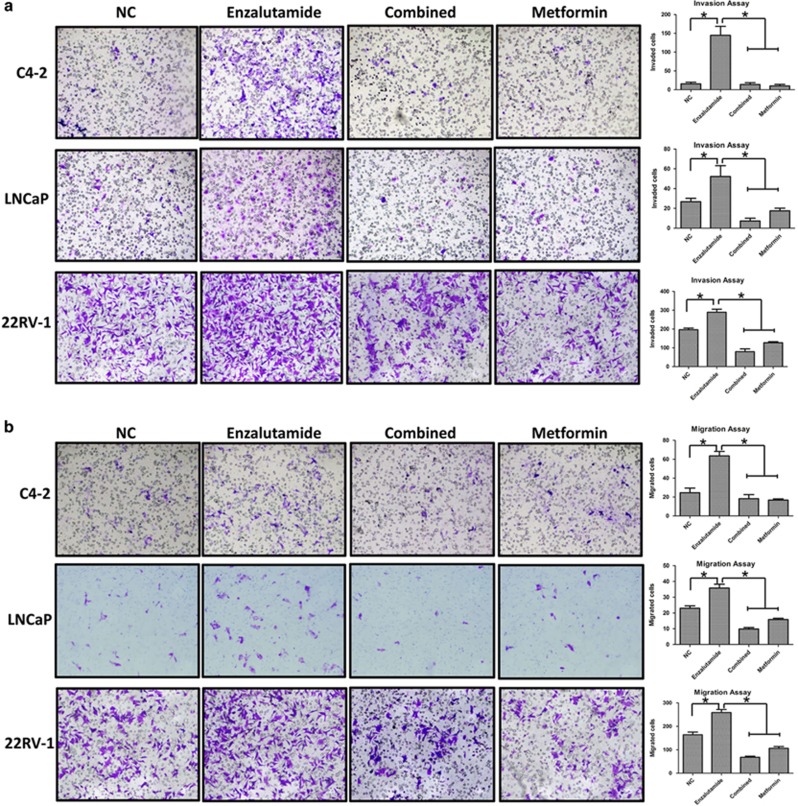
Metformin inhibits enzalutamide-induced cell invasion and migration. (**a** and **b**) C4-2, CWR22Rv1 and LNCaP cells were seeded in 24-well transwell chambers with or without Matrigel in growth media with or without enzalutamide (20 *μ*M) and metformin (5 mM) and cultured for 48 h. Cell invasion (**a**) and migration (**b**) were estimated. Quantifications were shown on right. The data represent means±S.D. *P<0.05

Multiple lines of evidence suggest that enzalutamide can promote cell invasion^28^ and metastasis.^29^ To determine whether metformin can antagonize this effect, we measured the invasion and migration of different prostate cancer cells using transwell assays with or without a Matrigel-coated membrane. Enzalutamide treatment alone indeed enhanced both invasion and migration in all three cell lines tested (Figures 2a and b). Similar to that reported previously^30^ that metformin is capable of inhibiting invasion and migration of PC-3 and CWR22Rv1 cells, we found that metformin inhibited the invasion and migration of both LNCaP and C4-2 cells (Figures 2a and b). More importantly, metformin is capable of inhibiting enzalutamide-induced invasion and migration of all three prostate cancer cell lines (Figures 2a and b).

### Effect of metformin on enzalutamide-induced EMT

**Figure 3:**
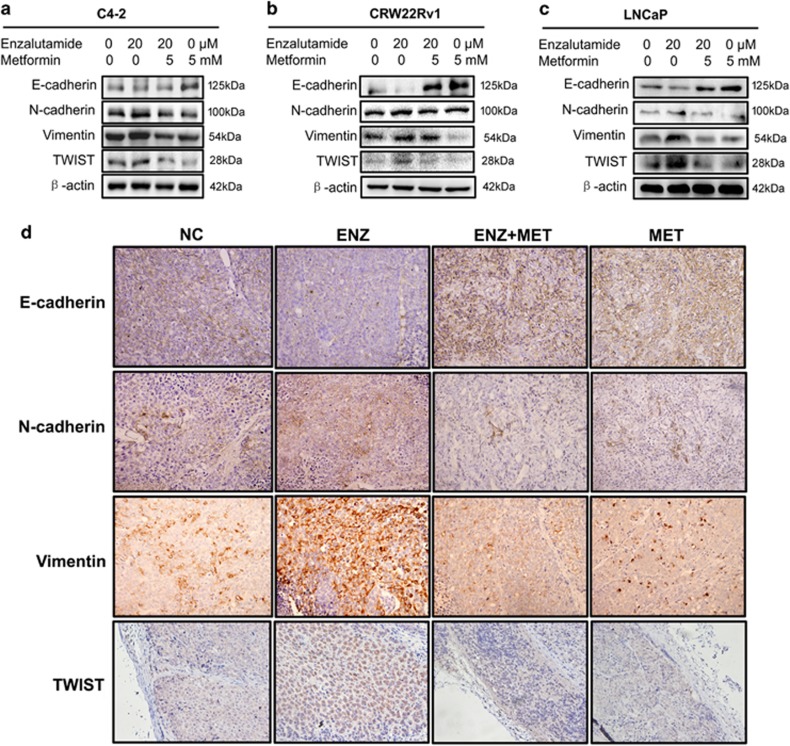
Metformin reverses enzalutamide-induced EMT. (**a–c**) C4-2, CWR22Rv1 and LNCaP cells was seeded in six-well plates and exposed to enzalutamide (20 *μ*M) or/and metformin (5 mM) for 48 h. Cell lysates were assayed by western blot with antibodies against E-cadherin, N-cadherin, Vimentin and Twist. (**d**) Tumors collected from 22RV1 cell xenograft model of nude mice treated as indicated were conducted IHC staining with antibodies against E-cadherin, N-cadherin, vimentin and twist. Magnifications: × 400

It has been reported that enzalutamide can promote prostate cancer metastasis via the TGF-*β*1/smad3/MMP9 axis.^28^ Since this axis plays important role in EMT, we decided to test whether enzalutamide can induce EMT and whether metformin can counteract enzalutamide-induced EMT. Three prostate cancer cells C4-2, CWR22Rv1, and LNCaP were treated with enzalutamide or metformin alone, or combination of both for 48 h followed by western blot assays to estimate the levels of factors involved in EMT. Figures 3a–c showed that enzalutamide treatment alone upregulated the levels of the mesenchymal biomarkers (N-cadherin, Vimentin and TWIST) and downregulated the epithelial markers (E-cadherin), suggesting enzalutamide is capable of promoting EMT. On the other hand, metformin is capable of counteracting enzalutamide-mediated up- and downregulating of the factors involved in EMT (Figures 3a–c). To quantify the expression of EMT-associated proteins, we used ‘Image J’ to analyze bands in western blots, the results of statistical analysis were showed in Supplementary Figure S1. Furthermore, these effects have been further substantiated by the IHC results in the CWR22Rv1-derived xenografts (Figure 3d).

### Metformin represses enzalutamide-induced STAT3 activation and TGF-*β*1 expression

**Figure 4:**
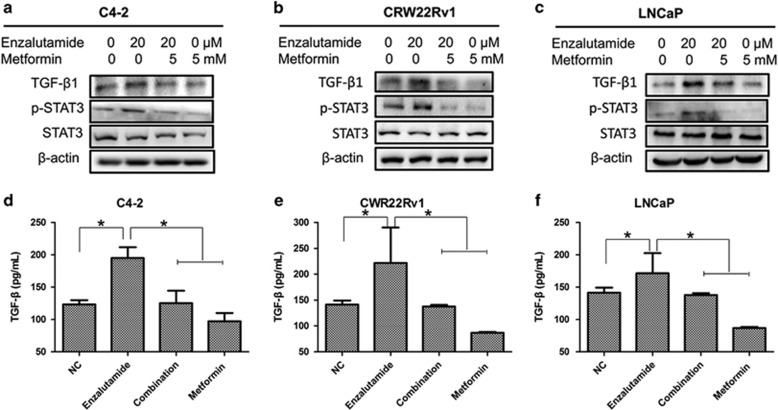
Metformin represses enzalutamide-induced TGF-*β*1 expression and STAT3 activation. (**a–c**) C4-2, CWR22Rv1 and LNCaP cells was seeded in 6-well plates and treated as indicated for 48 h. The levels of TGF-*β*1, p-STAT3 and STAT3 were estimated by western blot assays. (**d**–**f**) and the culture media were collect for ELISA assays of TGF-*β*1. The data represent means±S.D. *P<0.05

**Figure 5:**
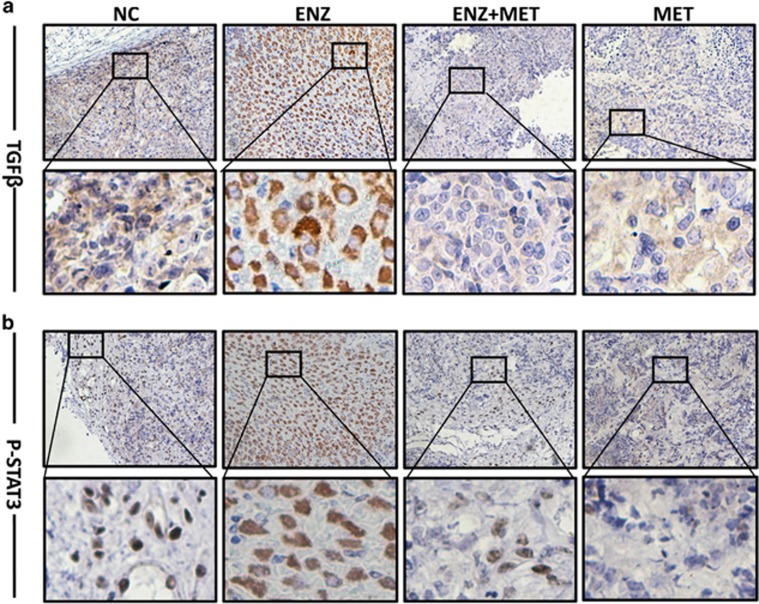
Tumor tissues from 22RV1 cell xenograft model of nude mice were stained for TGF-*β*1 (**a**) and p-STAT3 (**b**). Magnifications: × 400; higher magnification images, bottom panels

**Figure 6:**
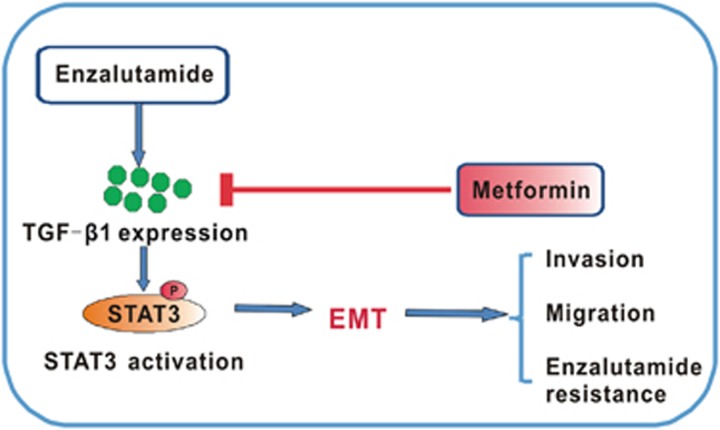
Schematic model of the hypothesized mechanism by which metformin reverses enzalutamide resistance

Since TGF-*β*1 plays an important role in the enzalutamide-induced invasion and metastasis^28^ and metformin is capable of inhibiting TGF-*β*1 by activating AMPK in both breast cancer patients and mouse model,^31^ we decided to determine if metformin counteracts enzalutamide-induced EMT in prostate cancer by downregulating TGF-*β*1. Western blot assays showed that enzalutamide upregulated the levels TGF-*β*1 in all three tested prostate cancer cell lines (Figures 4a–c) and metformin is able to counteract enzalutamide upregulated TGF-*β*1. These findings are consistent with the levels of TGF-*β*1 in culture media estimated by ELISA assays (Figures 4d–f). Finally, results from IHC staining of the xenografts derived from CWR22v1 cells (Figure 5a) further substantiated above-mentioned findings. Since STAT3 not only regulates a broad range of genes^32^ involved in EMT in different cancers^33^ but activated STAT3 also has been shown to confer enzalutamide resistance,^7, 34^ we then examined the effect of metformin on STAT3 activation. First, enzalutamide is capable of activating STAT3 in all three prostate cancer cell lines evidenced by the elevated levels of phosphorylated STAT3 (pSTAT3) without affecting the total levels of STAT3 (Figures 4a–c). More importantly, metformin is able to counteract enzalutamide-mediated STAT3 activation (Figures 4a–c). These findings were further substantiated by IHC staining of the CW22RV1 xenografts (Figure 5b). To further substantiate the inhibitory effect of metformin on enzalutamide-induced TGF-*β*1/STAT3 axis-regulated EMT, we used different concentrations of metformin (1, 5, 10 and 20 mM) combined with enzalutamide (20 *μ*M) to treat the three cell lines. We found that metformin was indeed capable of inhibiting enzalutamide-induced EMT via TGF-*β*1/STAT3 axis in a dose dependent manner (Supplementary Figure S2). Altogether, these results indicate that metformin can reverse enzalutamide resistance by counteracting enzalutamide-mediated STAT3 activation, subsequent upregulation of TGF-*β*1 and ultimate prostate cancer invasion and metastasis (Figure 6), suggesting that combination of metformin and enzalutamide could be more efficient in treating castration-resistant prostate cancer.

## Discussion

As a second-generation AR antagonist, enzalutamide has been widely used in treatment of advanced prostate cancer although the development of resistance is inevitable.^35^ In this study, we showed that EMT plays an important role in the development of enzalutamide resistance and metformin is capable of reversing enzalutamide-induced EMT. Mechanistically, we demonstrated that metformin exerts its effect by inhibiting TGF-*β*1 expression and STAT3 activation. These findings were highly consistent in our prostate cancer cell models *in vitro* and CWR22Rv1 xenograft mice models *in vivo*, suggesting that combination of metformin and enzalutamide would be more efficient in treating patients with advanced prostate cancer, especially the ones with enzalutamide-resistant cancer.

It is well established that alteration of TGF-*β*1 signaling and aberrant activation of STAT3 are closely correlated with the progression of various types of tumors, and an active STAT3 signaling pathway is essential for TGF-*β*-induced EMT.^36, 37, 38^ By directly binding to the promoter region of Snail, STAT3 serves as a regulatory transcriptional factor in regulation of the expression of Snail and subsequent induction of EMT.^39^ We have reported that activation of STAT3 is associated with EMT^30^ and CSCs repopulations.^40^ In this study, we found that metformin is capable of inhibiting enzalutamide-induced TGF-*β*1 expression and STAT3 activation. Based on that (1) elevated levels of TGF-*β*1 in enzalutamide-treated prostate cancers occurred concurrently with activation of STAT3 and enhanced EMT; and (2) metformin is capable of downregulating TGF-*β*1, inhibiting STAT3 activation and more importantly the enzalutamide-induced EMT, we speculate that one of the mechanisms in metformin-mediated reversal of enzalutamide resistance is by targeting EMT via TGF-*β*1/STAT3 axis. It has also been reported that metformin is capable of inhibiting the interaction between TGF-*β*1 and its receptor as well as type II TGF-*β*1 receptor dimerization.^41^ However, whether metformin’s effect on the interaction between TGF-*β*1 and its receptor is unclear and worth of further examination.

In addition, both ligand binding domain (LBD) mutation of AR and formation of constitutively active AR variants, especially AR-V7^42, 43^ play crucial roles in the development of enzalutamide resistance. Chengfei *et al.*^44^ showed that chronical treatment of prostate cancer patient with enzalutamide upregulated the expression of AR variants. Antonarakis *et al.*^45^ found that presence of AR-V7 in CTCs prior treatment conferred the cancer cells’ resistance to both enzalutamide and abiraterone. In addition, one of our previous studies^26^ has demonstrated that metformin is capable of downregulating ARV7. Therefore, we do not exclude the possibility that metformin counteracts enzalutamide-induced resistance by targeting either the mutant full-length AR or AR variants. Since autophagy has been considered as a novel mechanism for CRPC cells to evade enzalutamide treatment^46^ and metformin can inhibit autophagy in prostate cancer cells via AMPK/mTOR signaling pathway, it is worthy to explore the effect of metformin along this axis.

In summary, we demonstrated that metformin is capable of improving the efficacy of enzalutamide treatment by inhibiting enzalutamide-induced EMT. This finding provides the rationale for using combination of enzalutamide and metformin as a more efficient therapeutic strategy in the treatment of advanced prostate cancer.

## Materials and methods

### Reagents and cell culture

Human prostate cancer cell lines (LNCaP, C4-2 and CWR22Rv1) were obtained from Cell Bank of Shanghai Institutes for biological Sciences (Chinese Academy of Sciences), and cultured in RPMI 1640 supplemented with 10% FBS (Gibco) at 37 °C with 5% CO_2_. Enzalutamide was kindly provided by Dr. Lei and metformin was purchased from Sigma.

### Cell viability assay

C4-2, CWR22Rv1 and LNCaP cells were seeded into 96-well plates and after grown to 60–75% confluence, were treated with vehicle control, enzalutamide, metformin, and combination in RPMI 1640 with 10% FBS for 48 h. The viabilities of the cells were evaluated using the Cell Counting Kit-8 (CCK8) assay. Briefly, at termination of exposure, cells were aspirated and rinsed with PBS then treated with 10 *μ*l/well CCK-8 for 2 h at 37 °C. Absorbance was measured at 450nm spectrophotometrically (Bio-Rad, Hercules, CA, USA).

### Clonogenic assays

C4-2, CWR22Rv1 or LNCaP cells were seeded in 6-well plates with 1000 cells per well and incubated at 37 °C in culture media containing either enzalutamide (20 *μ*M) and metformin (5 mM) alone or in combination for 14 days with the medium changed every 7 days. At the end of the experiment, cells were fixed with methanol, stained with crystal violet and the numbers of colonies were counted.

### Transwell assays

To assess cell migration and invasion *in vitro*, we used 24-well transwell chambers with or without Matrigel. LNCaP, C4-2 and CWR22Rv1 cells were trypsinized and seeded into the top chamber at a density of 5 × 10^4^ cells per well in 200 *μ*l Dulbecco’s modified Eagle’s medium containing metformin (5 mM), enzalutamide (20 *μ*M) or combination of metformin (5 mM) and enzalutamide (20 *μ*M). The outer chambers contained 800 *μ*l of medium (10% fetal calf serum). After incubation at 37 °C for 48 h, cells attached to the upper surface of the membrane were carefully removed with cotton swabs, whereas cells that reached the underside of the chamber were fixed with 10% formalin and stained with crystal violet for 3 min at room temperature and counted.

### Western blotting

LNCaP, C4-2 and CWR22Rv1 cells were seeded in six-well plates, 2 × 10^5^cells/well, treated with corresponding reagents, including metformin (5 mM), enzalutamide (20 *μ*M) or combination of metformin (5 mM) and enzalutamide (20 *μ*M). Cell lysates were separated on SDS-PAGE followed by western blotting assay as described previously^26, 47^with the following primary antibodies: E-cadherin (1:1000 Proteintech, Rosemont, IL, USA), N-cadherin (1:500 Proteintech), TWIST (1:500 Santa Cruz Biotechnology, Dallas, TX, USA), Vimentin (1:1000 Proteintech), STAT3 (1:2000 Abcam, Cambridge, MA, USA), p-STAT3 (Tyr705) (1:2000 Cell Signaling, Danvers, MA, USA), TGF-*β*1 (1:200 Proteintech) and *β*-actin(1:5000 Cell Signaling). Image J (NIH, USA) was used to quantify the expression of proteins.

### TGF-*β*1 ELISA assay

C4-2, CWR22Rv1 and LNCaP Cells were seeded in six-well plates and treated with enzalutamide (20 *μ*M), metformin (5 mM) or combination for 24 h. The supernatant was collected and TGF-*β*1 released to the culture media was measured using commercially available enzyme-linked immunosorbent assay (ELISA) kits from Proteintech.

### *In vivo* tumorigenesis assay

CWR22Rv1 cells (four million/50 *μ*l) mixed with matrigel (1:1) were injected subcutaneously into the flanks of 6–7 weeks castrated male nude mice. Three weeks after injection, tumor-bearing mice (tumor volumes were around 50–100 mm^3^) were randomized into four groups (with 4 mice in each group) and treated with vehicle control (NC group), or enzalutamide (25 mg/kg/day) (ENZ group), or metformin (300 mg/kg/day) (MET group), or combination of enzalutamide (25 mg/kg/day) and metformin (300 mg/kg/day) (ENZ+MET group) for 3 weeks via esophageal gavaging. After killing the mice, the transplanted tumors were weighed and then fixed in 10% formalin and paraffin embedded for immunostaining examination.

### Immunohistochemical staining

Transplanted tumor specimens were fixed in 10% formaldehyde solution and embedded in paraffin, and sections were mounted onto glass slides. The sections were then deparaffinized in xylene, re-hydrated through ethanol, and heated for 30 min to enhance the heat-induced antigen retrieval. To block non-specific reactions, slides were blocked in respective serum at 4 °C overnight. Primary antibodies against E-cadherin (1:500 Proteintech), N-cadherin (1:50 Proteintech), TWIST (1:50 Santa Cruz Biotechnology), Vimentin (1:100 Proteintech), p-STAT3 (Tyr705) (1:100 Cell Signaling), TGF-*β*1 (1:50 Proteintech) were used. Tissue sections were incubated with each antibody overnight at 4 °C, and then incubated with horseradish peroxidase-conjugated anti-rabbit IgG secondary antibodies. Slides were subsequently treated with a streptavidin-peroxidase reagent and incubated in phosphate-buffered salinediaminobenzidine and 1% hydrogen peroxide, followed by counterstaining with Mayer’s haematoxylin.

### Statistical analysis

GraphPad Prism 5.0 (San Diego, CA, USA) was used for all statistical analyses. Data were presented as means±S.D. Differences between individual groups were analyzed by one-way analysis of variance followed by the LSD procedure for comparison of means, a *P*-value of <0.05 was considered statistically significant.

## Publisher’s Note

Springer Nature remains neutral with regard to jurisdictional claims in published maps and institutional affiliations.
