# Supplementary figures and images for: Metformin reverses prostate cancer resistance to enzalutamide by targeting TGF-β1/STAT3 axis-regulated EMT
Source: Cell Death Dis. 2017 Aug 24;8(8):e3007–. doi: 10.1038/cddis.2017.417 (PMC5596596; doi:10.1038/cddis.2017.417)

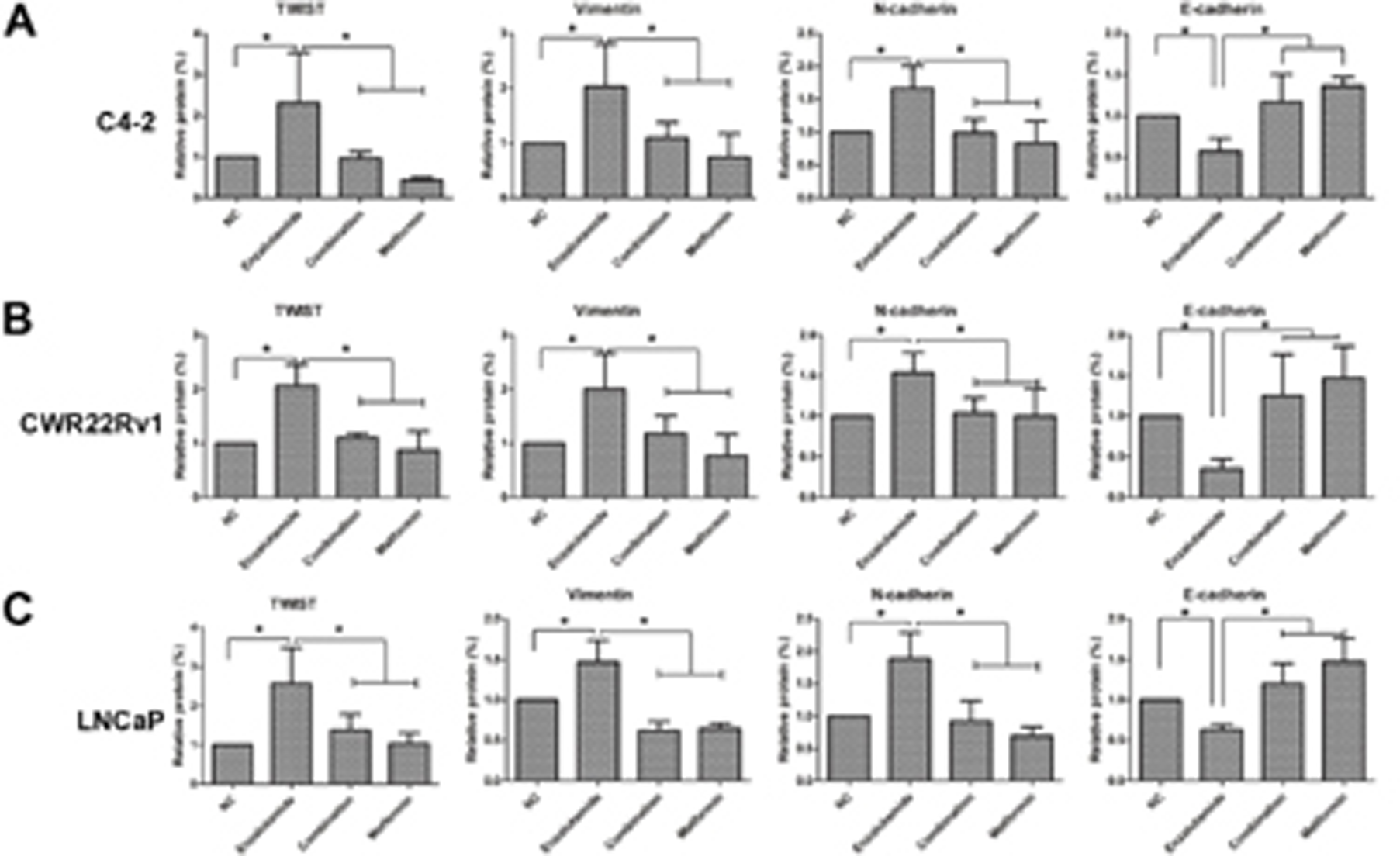

Supplement: Supplementary Figure S1 [file cddis2017417x1.tif]

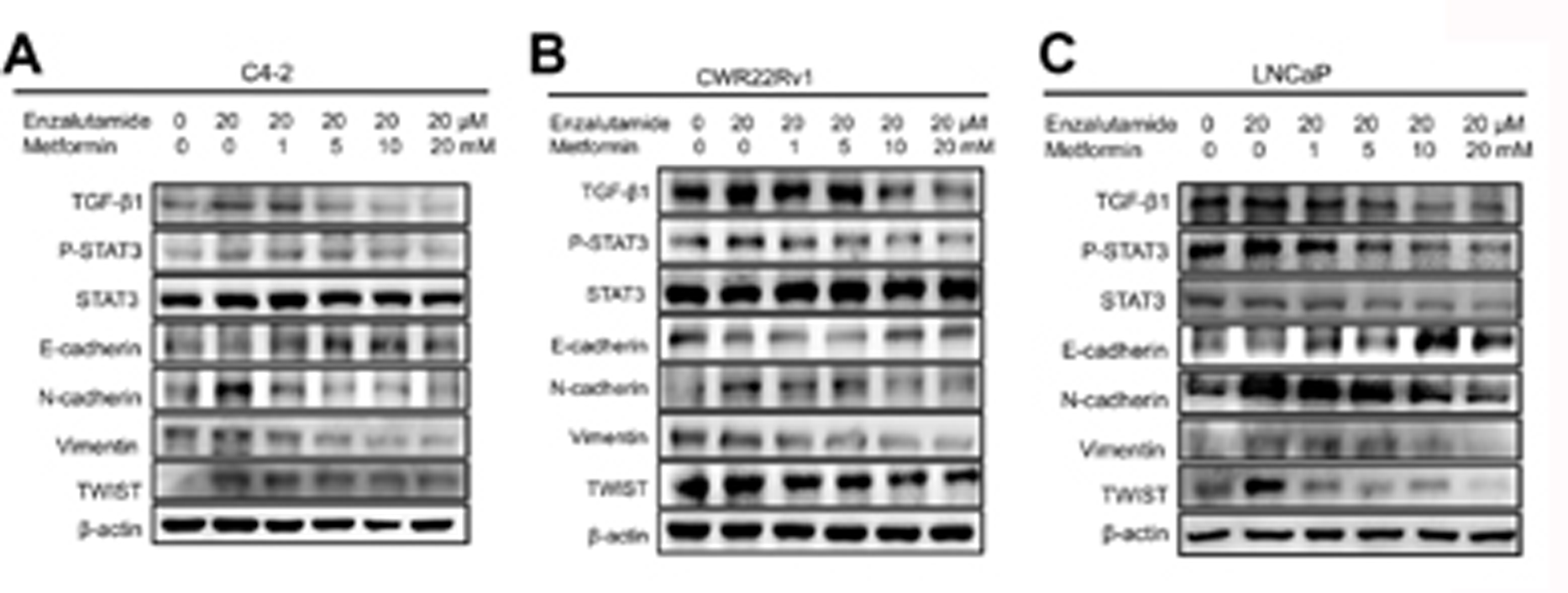

Supplement: Supplementary Figure S2 [file cddis2017417x2.tif]
