# Supplementary material for: Metformin reverses prostate cancer resistance to enzalutamide by targeting TGF-β1/STAT3 axis-regulated EMT
Source: Cell Death Dis. 2017 Aug 24;8(8):e3007–. doi: 10.1038/cddis.2017.417 (PMC5596596; doi:10.1038/cddis.2017.417)
Supplement: Supplementary Figures Legend [file cddis2017417x3.docx]

**Supplementary Figure S1: The quantification of the expressions of EMT-associated proteins by using Image J. (A).** The quatification of the expressions of TWIST, vimentin, N-cadherin, and E-cadherin in C4-2 cell line. **(B)** The quatification of the expressions of TWIST, vimentin, N-cadherin, and E-cadherin in CWR22Rv1 cell line. **(C)** The quatification of the expressions of TWIST, vimentin, N-cadherin, and E-cadherin in LNCaP cell line. The data represent means ± S.D. * P<0.05.

**Supplementary Figure S2: Metformin inhibits enzalutamide-induced TGF-β1/STAT3 axis-regulated EMT in a dose-dependent manner. (A-C)** C4-2, CWR22Rv1 and LNCaP cells was seeded in 6-well plates and exposed to enzalutamide (20 µM) or/and metformin (1Mm, 5mM, 10Mm, and 20mM) for 48h. Cell lysates were assayed by western blot with antibodies against TGF-β1, p-STAT3, STAT3, E-cadherin, N-cadherin, Vimentin and Twist.
